# A Resource Model of Team Resilience Capacity and Learning

**DOI:** 10.1177/10596011211018008

**Published:** 2021-05-12

**Authors:** Kyle M. Brykman, Danielle D. King

**Affiliations:** 1 Odette School of Business, 8637 University of Windsor, Windsor, ON, Canada; 2 3990 Rice University, Houston, TX, USA

**Keywords:** team resilience, conservation of resources theory, voice climate, team learning, learning goal orientation, information elaboration

## Abstract

A team’s capacity to bounce back from adversities or setbacks (i.e., team resilience capacity) is increasingly valuable in today’s complex business environment. To enhance our understanding of the antecedents and consequences of team resilience capacity, we develop and empirically test a resource-based model that delineates critical team inputs and outputs of resilience capacity. Drawing from conservation of resources theory, we propose that voice climate is a critical resource that builds team resilience capacity by encouraging intrateam communication and that leader learning goal orientation (LGO) amplifies this relationship by orienting team discourse toward understanding and growing from challenges. In turn, we propose that team resilience capacity is positively related to team learning behaviors, as teams with a higher resilience capacity are well-positioned to invest their resources into learning activities, and that team information elaboration amplifies this relationship by facilitating resource exchange. Results of a time-lagged, multisource field study involving 48 teams from five Canadian technology start-ups supported this moderated-mediated model. Specifically, voice climate was positively related to team resilience capacity, with leader LGO amplifying this effect. Further, team resilience capacity was positively related to team learning behaviors, with information elaboration amplifying this effect. Altogether, we advance theory and practice on team resilience by offering empirical support on what builds team resilience capacity (voice climate) and what teams with a high resilience capacity do (learning), along with the conditions under which these relationships are enhanced (higher leader LGO and team information elaboration).

As organizations increasingly structure work in teams (Bell, Kozlowski, & Blawath, 2012; Kozlowski & Ilgen, 2006), and teams encounter challenges that impair coordination and performance (Alliger, Cerasoli, Tannenbaum, & Vessey, 2015; King, Newman, & Luthans, 2016), it is important for scholars to explore and explain how teams develop a capacity needed to overcome the inevitable adversities that they face. Team resilience, defined as an emergent state reflecting a team’s *capacity* to bounce back from adversities or setbacks (Stoverink, Kirkman, Mistry, & Rosen, 2020), offers a valuable multilevel foundation to bridge insights from the individual and organizational paradigms, thereby developing a more complete understanding of resilience at work (Hartmann, Weiss, Newman, & Hoegl, 2020; Hartwig, Clarke, Johnson, & Willis, 2020). Although scholarly interest in team resilience is rapidly growing, current research is largely conceptual or restricted to extreme teams; thus, we still know surprisingly little about what builds resilience capacity in typical work teams, as well as the outcomes of this capacity (Duchek, 2020; Hartmann, Weiss, Newman, et al., 2020; King et al., 2016; Stoverink et al., 2020). Accordingly, the objective of this research was to answer three pressing questions: (a) what factors build team resilience capacity? (b) how does team resilience capacity relate to team learning behaviors? and (c) what leadership characteristics and/or team behaviors amplify these relationships?

We address these questions by drawing from conservation of resources theory (COR; Hobfoll, 1989, 2001) to develop and test a resource-based model of team resilience capacity (see Figure 1). COR is a valuable explanatory framework to understand how team resilience capacity is a linking pin between team inputs and outputs (Bowers, Kreutzer, Cannon-Bowers, & Lamb, 2017; Mathieu, Maynard, Rapp, & Gilson, 2008) because it describes the acquisition (input) and deployment (output) of core team resources to achieve team goals and proactively buffer against threats (Bardoel, Pettit, De Cieri, & McMillan, 2014; Stoverink et al., 2020).^1^ Indeed, several scholars have emphasized the utility of COR for explaining the emergence and function of team resilience (see Hartmann, Weiss, Newman, et al., 2020; Hartwig et al., 2020; King et al., 2016; Stoverink et al., 2020). Accordingly, we present a model that connects a specific team resource (voice climate) to a critical team output (learning) via team resilience capacity, along with moderators that qualify these effects, thereby elucidating some of the antecedents, outcomes, and boundary conditions of team resilience capacity.

**Figure 1.**
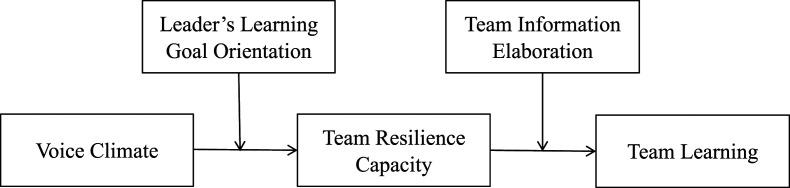
Resource model of team resilience capacity.

More specifically, our model proposes voice climate (shared perceptions within a team of the extent to which voice is encouraged; Morrison, Wheeler-Smith, & Kamdar, 2011) as a resource that builds team resilience capacity by encouraging open discourse, which is essential for helping teams manage and overcome future adversities. We further posit that leader learning goal orientation (LGO; a goal orientation focused on developing new skills and increasing competence; Dweck, 1986) activates and amplifies this relationship by orienting team discourse toward learning and growing from challenges and mistakes, thereby increasing the positive effects of voice climate on team resilience capacity. In turn, we argue that resilient teams—those with a high capacity for resilience—are well-positioned to invest their stocks of resources into learning activities (team members’ knowledge processing behaviors that enable team improvements; Harvey, Leblanc, & Cronin, 2019). Finally, we posit that information elaboration (an iterative team process of exchanging, discussing, and integrating ideas and information; Homan, van Knippenberg, Van Kleef, & De Dreu, 2007) amplifies this relationship by enhancing the mobilization of team resilience capacity via fluid information exchange and integration. Leveraging COR in this framework offers much-needed continuity to the field because it aligns to the dominant conceptualization of team resilience as an emergent capacity that is theorized to mediate the relationship between other team characteristics, states, behaviors, and outputs (Bowers et al., 2017; Hartmann, Weiss, Newman, et al., 2020; Stoverink et al., 2020). We assessed this model with a multisource, multi-wave field study involving 48 teams from five Canadian technology start-ups.

Altogether, our research addresses several calls in the literature by empirically evaluating the mechanisms of COR theory to explain what builds team resilience capacity (voice climate) and what teams with a high resilience capacity do (learning), along with leadership characteristics (LGO) and team behaviors (information elaboration) that enhance these relationships (Duchek, 2020; Hartmann, Weiss, Newman, et al., 2020; Stoverink et al., 2020). Accordingly, we advance theory and research on team resilience in several important ways. First, although scholars have theorized that learning is a core antecedent, component, and/or outcome of team resilience (e.g., Bowers et al., 2017; Stoverink et al., 2020; Sutcliffe & Vogus, 2003), we are unaware of any empirical research that has actually linked team resilience to learning. Rather, existing empirical research has primarily focused on performance and well-being outcomes (Gucciardi et al., 2018). This is surprising considering that learning is a more proximal outcome of team resilience than performance (Bell et al., 2012; Mathieu et al., 2008) and thus may help to explain why resilient teams tend to achieve positive adaptation and stronger performance. It is also problematic, as scant empirical attention to the links between team resilience and learning has helped to perpetuate the assumption that teams primarily learn *from* adversity, which fits the narrative of resilience as a process, but overlooks how resilient teams may be equally likely to engage in learning behaviors to anticipate and prepare for future challenges (Alliger et al., 2015; Duchek, 2020). Overall, we are unaware of any empirical research that has examined whether, why, or how team resilience relates to team learning.

Second, we focus on expanding the nomological network of team resilience capacity to include voice climate, leader LGO, team learning, and information elaboration. In doing so, we offer precision on the nuanced ways that specific team states (voice climate) affect team resilience capacity, juxtaposed to general contextual factors (e.g., psychological safety). Moreover, we focus on two moderators involving the emergence (leader LGO) and function (team information elaboration) of team resilience capacity, thereby clarifying the conditions under which (a) teams are more likely to develop a high resilience capacity and (b) teams with a high resilience capacity are more likely to engage in learning activities. As scholars have called for greater consideration of context in organizational behavior research (see Johns, 2006), we believe this integrated consideration of climate, leadership, and team processes and outcomes offers important advancements to the field. Finally, we also offer a multilevel perspective on team resilience, along with practical insights for leaders on how to build team resilience capacity, by accounting for the fundamental role of leadership in this model, thereby addressing calls for research that clarifies how leaders can facilitate productive sensemaking and promote resilience in teams (Alliger et al., 2015; Williams, Gruber, Sutcliffe, Shepherd, & Zhao, 2017).

## Theoretical Framework and Hypotheses

### A COR Model of Team Resilience

The fundamental principle of COR theory is that people strive to obtain and retain valued resources to assist with goal achievement (Hobfoll, 1989, 2001). Resources denote “objects, personal characteristics, conditions, or energies that are valued in their own right, or that are valued because they act as conduits to the achievement or protection of valued resources” (Hobfoll, 2001, p. 339). Hobfoll (1989) and colleagues (Halbesleben, Neveu, Paustian-Underdahl, & Westman, 2014; Hobfoll, Halbesleben, Neveu, & Westman, 2018) further assert that resources travel in packs or “resource caravans,” which denote pools of resources that come from the same environment—an important feature that we return to later. Another principle of COR theory is that those with more resources are less vulnerable to resource loss and more capable of resource gain (Chen, Westman, & Hobfoll, 2015; Hobfoll et al., 2018) and thus people are motivated to acquire resources to protect themselves against resource threats. This principle reflects the notion of “rich getting richer,” or “resource-gain spirals,” as people with more resources are able to invest their greater stocks of resources into activities that increase their available pool of resources (Bardoel et al., 2014). In sum, Hobfoll et al. (2018, p. 107) assert that “resource possession and lack thereof are integral to vulnerability and resilience.”

As noted earlier, COR theory has frequently been discussed as a potentially fitting and useful framework to understand the antecedents and consequences of team resilience (e.g., Hobfoll, Stevens, & Zalta, 2015; King et al., 2016; Stoverink et al., 2020). COR is especially applicable to understanding team resilience *capacity* because this conceptualization frames resilience as a team property that develops from other team experiences (inputs) to subsequently influence team behaviors (outputs; cf. Stoverink et al., 2020). Specifically, COR theory suggests that team resilience capacity emerges from environments that are “(a) rich in personal, social, materials, and energy resources, (b) allow access to those resources, and (c) provide safety and protection against resource loss and promote resource growth” (Hobfoll et al., 2015, p. 176). Thus, we draw from COR theory to develop a resource model that connects a caravan of important protective and promotive team resources, voice climate and leader LGO, to team learning via resilience capacity. That is, as described in greater detail below, we position voice climate as an important social resource that builds team resilience capacity by encouraging open communication and leader LGO as an additional team resource that amplifies the positive effect of voice climate on team resilience capacity by activating growth-oriented attitudes toward adversity and orienting team discourse toward positive views of mistakes intended for growth. Next, we argue that team resilience capacity affects team learning such that teams high on resilience capacity invest their abundant stocks of resources into learning activities to further enhance and protect their resources and specify team information elaboration as a resource mobilization mechanism that augments the benefits of team resilience capacity for learning via efficient interpersonal exchange (i.e., “crossover”; Bolger, DeLongis, Kessler, & Wethington, 1989; Hobfoll et al., 2018; Stoverink et al., 2020). Before elaborating on this model, we first describe our conceptualization of team resilience capacity to clarify our perspective.

### Conceptualizing Team Resilience Capacity

While research on team resilience is rapidly growing, different scholars have adopted different conceptualizations, which has resulted in a somewhat fragmented body of research. As has been noted elsewhere (Duchek, 2020; Hartmann, Weiss, & Hoegl, 2020; Hartwig et al., 2020; Stoverink et al., 2020), team resilience is commonly conceptualized as either a capacity, process, or outcome. That is, scholars have either defined team resilience as (a) an emergent state denoting a team’s capacity to bounce back from future setbacks, (b) a dynamic social process that enables positive adaptation to collectively experienced threats or challenges, or (c) the demonstration of resilience as manifested in positive outcomes (e.g., recovery and growth) after an adversity.

Our perspective is that all of these approaches are appropriate. However, it is critical for scholars to be clear and precise regarding their chosen conceptualization to ensure a unified approach to understanding this phenomenon. To follow this advice, we explicitly conceptualize and define team resilience as a team *capacity* to bounce back from adversities or setbacks and reserve the term “resilient teams” to denote teams with a high capacity for resilience. We also use the term “team resilience capacity” throughout to clarify this focus, whereas we use the term “team resilience” to refer to the literature and/or phenomenon more broadly. This conceptualization of team resilience as an emergent capacity has become a dominant approach in the literature, especially for quantitative research (e.g., Bowers et al., 2017; Maynard & Kennedy, 2016; Stephens, Heaphy, Carmeli, Spreitzer, & Dutton, 2013; Stoverink et al., 2020). Operationally, it reflects team members’ shared beliefs in, or perceptions of, their collective capacity to overcome future adversities or setbacks (Carmeli, Friedman, & Tishler, 2013; Hartmann, Weiss, Newman, et al., 2020; Vera, Rodríguez-Sánchez, & Salanova, 2017).

It is also important to clarify the role of adversity within this conceptualization. We define adversity as “challenging events and circumstances [that] place stress on individuals and on team processes” (Alliger et al., 2015, p. 177), including, for example, difficult assignments, time pressure, insufficient resources, and conflict. Adversity can range from chronic to acute, short to extended, and sudden to gradual onset (Williams et al., 2017). It has the potential to harm team performance because it often impairs team coordination and goal attainment (Stoverink et al., 2020). Although adversity is an essential component for teams to *demonstrate* resilience (by overcoming the adversity), several scholars have explained why it is not a prerequisite for teams to develop a high capacity for resilience. For example, Hartmann, Weiss, and Hoegl (2020, p. 45) argue: “Team resilience capacity describes the potential of a team to show positive adaptation if and when the team faces adverse circumstances… Teams may hold this capacity regardless of whether they have ever faced or will ever face a setback or adversity” (see also Stoverink et al., 2020). Thus, while an adversity experience is a defining element of the team resilience process, and a necessary precondition for a team to demonstrate resilience, teams need not experience adversity to develop a capacity to overcome future adversities, nor to harness this capacity to engage in proactive learning behaviors (Hartmann, Weiss, & Hoegl, 2020; Stoverink et al., 2020). With this foundation in place, we elaborate on each proposition in greater detail below.

### Voice Climate and Team Resilience Capacity

Employee voice—discretionary communication of information, ideas, or issues that may be challenging in nature but is intended for improvement (Morrison, 2011)—is a valuable team behavior that is positively related to team performance (e.g., Frazier & Bowler, 2015), learning (e.g., Edmondson, 1999), and innovation (e.g., Guzman & Espejo, 2019). Given that employees are generally reluctant to speak up with ideas and concerns (Detert & Edmondson, 2011), recent research has emphasized the importance of *voice climate*—shared team perceptions of the extent to which voice is encouraged on the team (Morrison et al., 2011)—for stimulating voice, thereby ensuring that organizations reap their collective benefits (Frazier & Bowler, 2015). In line with individual-level research (Ashford, Rothbard, Piderit, & Dutton, 1998), the primary beliefs that underlie voice climate are (a) voice safety—shared belief about whether speaking up is safe versus dangerous—and (b) voice efficacy—shared belief about whether group members are able to speak up effectively and their input is taken seriously (Morrison et al., 2011). In the present research, we position voice climate as a critical resource that builds team resilience capacity by ensuring that team members feel safe and capable of vocalizing pertinent information, high-quality ideas, and impending concerns, which is integral to build their capacity to navigate and overcome future adversities.

Theoretical models and empirical insights support the potential for voice climate to foster team resilience capacity. For example, Gucciardi et al. (2018) highlight the role of supportive team norms for building team resilience, as norms provide important information that guide team approaches and responses to adversity. Of particular relevance to our model, Stoverink et al. (2020) drew from COR theory to identify several factors that build team resilience capacity, including team potency and psychological safety. They theorized that these states provide necessary resources that enable teams to manage adversities by vocalizing problems and forming shared understandings. Similarly, Bowers et al. (2017) modeled team resilience as a second-order emergent state that results from inputs such as psychological safety and collective efficacy. Voice climate shares similar features with these constructs; however, it is a more specific team state that involves the combination of safety and efficacy beliefs, and focuses on intrateam communications, rather than other risky behaviors (Morrison et al., 2011).^2^ Thus, in line with COR theory, voice climate is particularly relevant for facilitating team resilience capacity because it facilitates resource acquisition (efficacy) and protects against resource loss (safety).

Empirical findings also support the potential for voice climate to influence team resilience capacity based on the value of open, trusting team communications. For example, Vera et al. (2017) found that teamwork (e.g., respectful interactions) builds team resilience capacity. Related research also demonstrates the importance of team communication for shaping team resilience, such as by generating new ideas and creating alignment within the team (Carmeli et al., 2013; Gomes, Borges, Huber, & Carvalho, 2014). More recently, Li and Tangirala (forthcoming) found that team promotive and prohibitive voice, proximal outcomes of voice climate (Frazier & Bowler, 2015; Morrison et al., 2011), enable process innovation (resource acquisition) and error management (resource protection) in response to major organizational change events. Altogether, prior research suggests that voice climate is a central mechanism for building team resilience capacity.

Drawing from COR theory (Hobfoll, 1989), we propose that voice climate provides teams with a necessary environmental resource of safety and efficacy to voice, which increases their capacity to overcome future challenges via resource acquisition and protection. For example, voice climate can help prevent rigid responses to difficulties by encouraging open discourse before adversity strikes, thereby preparing teams to manage difficult and unexpected events (Maynard & Kennedy, 2016; Sutcliffe & Vogus, 2003). As Alliger et al. (2015, p. 179) elaborate, resilient teams “vocalize concerns and give one another a ‘heads-up’ when they see a challenge looming. They are particularly good at attending to unfavorable information and are careful not to dismiss concerns prematurely.” Thus, relative to teams with a low voice climate, we expect teams with a high voice climate will develop a greater capacity to overcome future adversities. Therefore, we propose

**Hypothesis 1:** Voice climate is positively related to team resilience capacity.

### The Moderating Effects of Leader LGO

As noted above, in applying COR theory to the study of resilience, Hobfoll et al. (2015, p. 176) argue that the capacity for resilience emerges from “resource rich” environments that “provide safety and protection against resource loss and promote resource growth.” They further assert that resources exist in “caravans” from the same environment, such as how teams with a positive climate tend to also have supportive leadership and decision-making autonomy, and that each additional personal, social, and/or material resource further augments an entity’s capacity for resilience (Hobfoll et al., 2015, 2018; see also Halbesleben et al., 2014). To account for this dynamic, we consider the role of an additional team resource, leader LGO, for activating and amplifying the constructive effects of voice climate on team resilience capacity by orienting voice climate toward discussions of challenges and learning from mistakes, as opposed to other voice content (e.g., novel ideas) less relevant to resilience. Importantly, leader LGO also aligns to environmental properties conducive for the development of team resilience capacity based on COR theory (Hobfoll et al., 2015) in that it has the potential to facilitate both resource acquisition (focusing goals on competency development) and resource protection (offering latitude for team members to make mistakes).

LGO is characterized by investment in goal-directed efforts with the intention of developing new skills and increasing competence (Dweck, 1986). Individuals with a high LGO thus tend to feel energized by challenges and hold constructive views of mistakes as a means for growth (Dweck, 1986; Vandewalle, Nerstad, & Dysvik, 2019). As leaders hold power and prominence in team hierarchies, with a primary function of guiding their team toward shared goals (Northouse, 2021; Piccolo & Buengeler, 2013), their personal goal orientation has the potential to dramatically shape their teams’ expectations and subsequent behaviors (Kozlowski & Ilgen, 2006; Mathieu et al., 2008). For example, leaders with a high LGO would tend to encourage team members to pursue difficult tasks, frame challenges as a learning opportunity, and foster discussions focused on skill development (Dragoni, 2005; Dragoni & Kuenzi, 2012). Indeed, broader research documents how leaders’ personal characteristics tend to affect team members’ behaviors via indirect (e.g., modeling) and direct (e.g., goal-setting) mechanisms that signal collective expectations (cf. Dragoni & Kuenzi, 2012; Piccolo & Buengeler, 2013). In particular, the trickle-down effect of leadership (Johnson et al., 2017; Mayer, Kuenzi, Greenbaum, Bardes, & Salvador, 2009), which is grounded in social learning theory (Bandura, 1977), details that leader characteristics often “trickle-down” to influence followers’ cognitions and behaviors. In support of this phenomenon, Dragoni and Kuenzi (2012) found that leader goal orientation indirectly affects team performance via unit goal orientation, and Zhu and Akhtar (2019) found that leader LGO indirectly affects employee behavior via leader openness, both of which show how leader characteristics, specifically goal orientation, trickle-down to affect team behaviors.

Accordingly, in shaping team members’ work approaches, we argue that leader LGO activates and amplifies the constructive effects of voice climate on team resilience capacity by orienting team communications toward developing competencies through challenging work (resource acquisition) and learning from mistakes in the pursuit of growth (resource protection). Stated otherwise, voice climate is a social resource that builds team resilience capacity via encouragement to speak up, and LGO amplifies this effect by orienting team communications toward learning from mistakes, thereby activating the potential for voice climate to build team resilience capacity. By contrast, leaders with a low LGO are threatened by challenges and the prospect of failure, and thus, the positive effects of voice climate would be mitigated for their teams because their followers would be less likely to engage in open discourse focused on understanding and growing from mistakes, which would otherwise help them to fully leverage voice climate for building resilience capacity.

Theoretical and empirical insights indirectly support this argument. For example, Barton and Kahn (2018) noted that team members look to leaders to frame adversity experiences and model appropriate responses. Prior work has also demonstrated a link between transformational leadership and team resilience capacity effects via leaders converting crises into developmental challenges (Sommer, Howell, & Hadley, 2016; Vera et al., 2017). Here, we assert that leader LGO enhances the positive effect of voice climate on team resilience capacity by setting the tone for team members to view adversity as a challenge, rather than a hindrance, and by encouraging open discussions of growth-oriented perspectives to setbacks. Voice climate equips teams with a necessary condition to overcome potential future adversities via open discourse, and leader LGO combines with this climate to further build team resilience capacity by demonstrating that events requiring resilience are opportunities for learning and growth. Therefore, we propose

**Hypothesis 2:** Leader learning goal orientation amplifies the positive relationship between voice climate and team resilience capacity.

### Team Resilience Capacity and Team Learning

Drawing from Harvey et al. (2019), we define *team learning* as team members’ behaviors related to knowledge processing, which enables team improvements. Edmondson (1999) identified several core learning behaviors, including “asking questions, seeking feedback, experimenting, reflecting on results, and discussing errors or unexpected outcomes of action” (p. 353). Edmondson (1999) further specified that learning behaviors consume valuable resources (e.g., time and energy) without assurances of positive results, and thus, teams will only invest these resources into learning activities under positive team conditions (see also Harvey et al., 2019).

Therefore, in line with COR, we propose that teams with a high resilience capacity will engage in more learning than teams with a low resilience capacity because they have greater stocks of resources to invest into learning activities and can preserve and/or acquire more resources via learning. As Maynard and Kennedy (2016, p. 22) elaborate, “team resilience can provide adaptability to future threats by creating resources that can be drawn upon, combined, or molded to new situations as needed.” In that sense, teams with a high capacity for resilience are well-positioned to invest resources toward learning because it is a means to acquire more resources (e.g., new knowledge and shared understanding; Maynard & Kennedy, 2016; Sutcliffe & Vogus, 2003). Resilient teams do not strictly seek feedback and experiment during (e.g., manage and coping) or after adversity occurs (e.g., mend and adaptation), but also before adversity strikes (e.g., minimize and anticipation), thereby building resilient resources that enable them to overcome future challenges (Alliger et al., 2015; Duchek, 2020; Stoverink et al., 2020; Williams et al., 2017).

The importance of resilience for learning is deeply embedded within the broader resilience literature. For example, Tugade and Fredrickson (2004) argued that a core outcome of individual resilience is the capacity to learn from life’s setbacks. This insight likely explains Seery, Holman, and Silver’s (2010) and Seery, Leo, Lupien, Kondrak, and Almonte’s (2013) findings that individuals who experienced some lifetime adversity reported being more resilient than those who experienced no or high adversity, as they theorized that experiencing some adversity enabled individuals to learn effective coping skills, develop support networks, and feel a sense of mastery. This sentiment is further reflected in the post-traumatic growth concept, such that some individuals emerge stronger after trauma because they channeled difficulties into learning activities, including reflection, problem-focused coping, meaning-making, and changing worldviews (Tedeschi & Calhoun, 2004).

Several organizational scholars have also described learning as an outcome of team resilience capacity. For example, Barton and Kahn (2018) argued that resilient teams engage in “relational pauses,” a type of learning behavior focused on improving information processing and goal coordination. As well, Stoverink et al. (2020) argued that resilient teams engage in thoughtful reflection, knowledge crystallization, and information integration when adversity strikes. Sutcliffe and Vogus (2003) also suggested that resilient teams are more likely to accumulate knowledge and develop competencies because they are willing to make mistakes for developmental purposes and view setbacks as growth opportunities. Similarly, Bowers et al. (2017) argued that resilient teams learn from prior challenges because they prepare them to adapt to future ones. Therefore, we propose

**Hypothesis 3:** Team resilience capacity is positively related to team learning.

### The Moderating Effects of Team Information Elaboration

In addition to explaining how individuals protect, acquire, and preserve resources, COR theory elaborates on how resources are exchanged within teams via “crossover,” such that individual members’ experiences, emotions, and resources transfer within the social environment (Bolger et al., 1989; Hobfoll et al., 2018). This crossover model proposes that these mechanisms of resource exchange enable resilient teams to fully capitalize on their abundant pool of resources (Hobfoll et al., 2018). Accordingly, we position team information elaboration as a central mechanism that amplifies the effects of team resilience capacity on team learning by specifying the extent to which resilient teams mobilize their resources. *Information elaboration* denotes an iterative process of exchanging information and ideas, discussing and seeking clarification on these perspectives, and integrating this information, which helps teams capitalize on individual members’ discrete knowledge and skills (Homan et al., 2007; Resick, Murase, Randall, & DeChurch, 2014). Thus, it extends beyond information sharing to also capture the extent to which team members deeply reflect on and integrate each other’s perspectives and ideas.

Accordingly, we expect information elaboration to enhance the positive effects of team resilience capacity on learning, such that resilient teams engage in even more learning to the extent that they integrate diverse opinions and seek clarifications. This perspective maintains that even teams with a high capacity for resilience will struggle to learn before, during, or after adversity strikes if they fail to effectively exchange or elaborate on distributed information (Stoverink et al., 2020). Information elaboration is especially important for leveraging a team’s resilience capacity because adversity tends to narrow information processing and trigger anxiety, thereby undermining team coordination and communication (Barton & Kahn, 2018; Sutcliffe & Vogus, 2003; Waller, 1999). Empirical research also supports the amplifying role of information elaboration on the relationship between team resilience capacity and learning. For example, Rauter, Weiss, and Hoegl (2018) found that team reflexivity moderated the effects of team affective reactions to a setback on team learning, which suggests that the extent to which resilient teams engage in learning depends on whether team members reflexively share information. As well, using a scenario-based simulator training with military teams, Mjelde, Smith, Lunde, and Espevik (2016) demonstrated that closed-loop communication, whereby team members exchanged information and coordinated activities through a feedback process, was integral to team adaptation and performance during a crisis. Therefore, we propose

**Hypothesis 4:** Team information elaboration amplifies the positive relationship between team resilience capacity and team learning.

### Overall Moderated-Mediated Model

As described earlier, several scholars have conceptualized team resilience as a team-centric capacity that is theorized to mediate the relationship between other team states and outcomes (Bowers et al., 2017; Stoverink et al., 2020). Accordingly, our overall model suggests that team resilience capacity is a critical team resource that explains why voice climate relates to team learning and that leader LGO and team information elaboration sequentially moderate this mediated relationship. It is important to note that empirical research also supports a link between voice climate and learning (Edmondson, 1999; Li, Liao, Tangirala, & Firth, 2017), although the specific mechanism(s) linking these constructs is largely unspecified. One possibility is that voice climate facilitates voice behaviors (Frazier & Bowler, 2015; Morrison et al., 2011), which affects team learning by encouraging team members to seek clarifications and admit mistakes (e.g., Tangirala & Ramanujan, 2008). Alternatively, our model argues that team resilience capacity relates voice climate to team learning, such that voice climate builds a team’s capacity to overcome future adversities, which motives and enables team members to expend resources in the pursuit of learning (Alliger et al., 2015; Stoverink et al., 2020). Our model also proposes that leader LGO amplifies the positive effects of voice climate on team resilience capacity via trickle-down effects through which leaders’ positive views of challenges and growth transfer to the team and amplify the beneficial role of voice climate in enhancing team resilience capacity. In turn, we propose that information elaboration amplifies the positive effects of team resilience capacity on team learning via efficient resource mobilization. Therefore, we propose

**Hypothesis 5:** Team resilience capacity mediates the positive effects of voice climate on team learning.
**Hypothesis 6:** The mediated relationship between voice climate, team resilience capacity, and team learning is amplified by leader learning goal orientation (stage 1) and team information elaboration (stage 2).

## Method

### Sample and Procedures

We assessed the proposed model with a time-lagged, multisource field study involving 48 teams from five established Canadian technology start-ups. These organizations were in existence for at least 4 years (*μ* = 6 years) and ranged in size from 20 to 350 employees at the time of data collection. These teams worked in various functions, including engineering, marketing, and customer service. This context was relevant to our research because start-ups experience heightened failure rates (Headd, 2003), in which case it is particularly important for their teams to develop resilience capacity. At the same time, as noted above, these start-ups were fairly established and of considerable size and thus are more similar to typical organizations than emerging start-ups. Thus, teams in our sample likely faced similar challenges as teams in conventional organizations (e.g., member change; Alliger et al., 2015).

We first administered the team member survey, which included measures for voice climate and team resilience capacity. Two weeks later, we administered the leader survey, which included measures for leader LGO, team information elaboration, and team learning.^3^ This multisource approach enabled us to proactively address concerns of common-method bias, particularly between our independent and dependent variables (Podsakoff, MacKenzie, Lee, & Podsakoff, 2003; Podsakoff, MacKenzie, & Podsakoff, 2012). It also helped to increase confidence in the robustness of our results, such that they are not spurious artifacts due to teams holding positive perceptions of themselves overall.

As team membership has become increasingly fluid in modern organizations, we explicitly defined the boundaries for teams in this study through discussions with our partners, on the basis that the team members regularly interacted with each other, had shared goals, and reported to the same leader(s), who was responsible for managing team goals and performance (Chan, 1998; George, 1990). To ensure that participants reflected on their experiences with the appropriate team, we identified team membership at the beginning of each survey (with an organizational chart) and asked participants to complete the survey with this team in mind. Participants’ average team tenure was one and a half years, suggesting that they had ample shared experiences to develop resilience capacity.

To be eligible for the study, each team was required to consist of at least three members in addition to the team leader, as otherwise they more closely resemble a dyad than a team. At the same time, we included teams in our analysis who met this qualification, but in which only two members and a leader completed the surveys (*n* = 9). Although some scholars advocate for removing teams that fail to reach a prespecified number or proportion of responding team members due to issues of interrater agreement, removing teams on this basis introduces new problems because these teams may be different for important reasons related to our research questions, such as low engagement in voice climate, thereby creating a biased sample (cf. Allen, Stanley, Williams, & Ross, 2007; O’Neill, McLarnon, Hoffart, Woodley, & Allen, 2018). Consequently, several recent studies advocate against such deletion methods because, among other reasons, they reduce statistical power and distort effect sizes (Hirschfeld, Cole, Bernerth, & Rizzuto, 2013; Stanley, Allen, Williams, & Ross, 2011). Nevertheless, we conducted ANOVAs to compare data between these nine teams in which only two members and a leader completed the survey and teams in which three or more members and a leader responded (*n* = 39) and did not observe any discernable or significant differences.

In total, we distributed surveys to 72 teams, which were comprised of 462 team members and 74 team leaders. We received responses from 308 team members (67%) and 50 team leaders (69%). We removed 24 teams from our analysis because of insufficient data—either because less than two members participated (*n* = 2), the team leader did not participate (*n* = 20), or both (*n* = 2). Accordingly, our analysis was based on data from 48 teams, which were comprised of 215 team members and 50 team leaders.^4^ Of these team members, 48% identified as male. The dominant ethnicities were Caucasian (53%) and Asian (35%). Their average age was 30 years old (*SD* = 5.44), and 86% had at minimum a university degree. Their average organizational tenure was 2 years (*SD* = 1.72) and average team tenure was 1.5 years (*SD* = 1.15). Of these team leaders, 70% identified as male. The dominant ethnicities were Caucasian (58%) and Asian (26%). Their average age was 36 years old (*SD* = 6.11), and 72% had at minimum a university degree. Their average organizational tenure was 3.75 years (*SD* = 2.34), average team tenure was 2.15 years (*SD* = 1.95), and average managerial experience was 5.70 years (*SD* = 4.50).

### Measures

#### Team member survey

We measured voice climate with Frazier and Bowler’s (2015) 6-item scale. The scale prompt states, “Members of my team are encouraged to…” followed by the items, such as “develop and make recommendations concerning issues that affect the team” and “speak up and encourage others on the team to get involved in issues that affect the team.” We measured team resilience capacity with Stephens et al.’s (2013) 3-item measure by replacing the phrase “this TMT” (top management team) with “my team.” Example items include “my team knows how to cope with challenges” and “my team is able to cope with difficult periods of time.” Thus, both scales used a referent-shift approach (Chan, 1998), consistent with best practices on assessing shared team constructs (Hartmann, Weiss, Newman, et al., 2020). Importantly, this operationalization matches our conceptualization of team resilience capacity as reflecting team members’ shared beliefs in their collective capacity to overcome future adversities or setbacks.

#### Leader survey

We measured leader’s LGO with Vandewalle’s (1997) 4-item scale. Example items include “I am willing to select a challenging work assignment that I can learn a lot from” and “I enjoy challenging and difficult tasks at work where I’ll learn new skills.” We measured team information elaboration with van Dick, van Knippenberg, Hägele, Guillaume, and Brodbeck’s (2008) 7-item scale. Example items include “members of my team exchange a lot of information about our tasks” and “members of my team often say things that lead each other to learn something new.” Finally, we measured team learning with Edmondson’s (1999) ^5^ 7-item scale. Example items include “my team actively reviews its own progress and performance” and “my team relies on outdated information or ideas (reverse).” We anchored all team member and leader measures on 5-point Likert scales ranging from “Strongly Disagree” (1) to “Strongly Agree” (5).

## Analyses and Results

### Preliminary Testing

To begin, we evaluated the appropriateness of aggregating voice climate and team resilience to the team level. Both constructs exhibited ICC and *r*_wg(*J*)_ values above suggested cutoffs (James, Demaree, & Wolf, 1984; see Table 1), which implies high levels of within-team agreement and thus that these are suitable team-level variables. We also examined whether there were any nesting effects due to organizational membership to determine the requirement for multilevel modeling. That is, although all of the relationships were conceptualized at the team level, we collected data from five different organizations and it is possible that the constructs vary due to overarching organizational differences (Bliese, 2000). ANOVA results revealed that organizational membership did not significantly influence any of these variables: voice climate (*F* = .10, *n.s.*), team resilience capacity (*F* = 1.27, *n.s.*), leader LGO (*F* = .96, *n.s.*), team information elaboration (*F* = 1.69, *n.s.*), and team learning (*F* = .96, *n.s.*). As a result, we assessed all hypotheses at the team level, though we controlled for organizational membership to account for potential effects in our model. We also controlled for team size because prior research suggests that it can significantly affect team resilience capacity (e.g., Gomes et al., 2014). Table 2 lists means, standard deviations, and correlations for all variables in the model.

**Table 1.** Aggregation Statistics for Team-Level Variables.

Variable	F	*r*_*wg*(*J*)_ mean	ICC (1)	ICC (2)
1. Voice climate	2.64^**^	.94	.26	.61
2. Team resilience capacity	3.12^**^	.92	.15	.44

*Note. n* = 215. ^**^ *p* <.01. ICC = intraclass correlation coefficient.

**Table 2.** Descriptive Statistics and Correlations.

Variable	Mean	*SD*	1	2	3	4	5	6	7
1. Organization *(dummy code)*	3.00	2.28	—						
2. Team size	6.44	5.20	.25	—					
3. Voice climate	4.38	.41	.06	−.09	*(.87)*				
4. Leader LGO	4.46	.46	−.27	−.24	.13	*(.72)*			
5. Team resilience capacity	4.12	.51	.06	.02	.60^**^	.15	*(.91)*		
6. Information elaboration	3.83	.63	.11	.10	.22	−.16	.19	*(.88)*	
7. Team learning	4.11	.36	−.06	−.16	.50^**^	.22	.50^**^	.39^**^	*(.67)*

*Note. n* = 48. *SD* = standard deviation; LGO = learning goal orientation. Scale reliabilities are reported on the diagonal in parentheses. ^**^ *p* <.01.

### Hypotheses Testing

We centered all variables that defined a product term to clarify the regression coefficients and interpretation of the interactions (Dawson, 2014) and proceeded with hypothesis testing. In support of Hypothesis 1, we found that voice climate was positively related to team resilience capacity (β = .60, *p* <.01). As well, we found support for Hypothesis 2, as leader’s LGO moderated the effects of voice climate on team resilience capacity (β = .31, *p* <.05). We also found support for Hypotheses 3 and 4, as team resilience capacity was positively related to team learning (β = .50, *p* <.01) and team information elaboration moderated the effects of team resilience capacity on team learning (β = .29, *p* < .01). Hierarchical regression results are reported in Table 3.

**Table 3.** Hierarchical Regression Results for Team Resilience Capacity and Team Learning.

	Team resilience capacity				Team learning			
	Model 1	Model 2	Model 3	Model 4	Model 5	Model 6	Model 7	Model 8
Control variables								
Organization	.06	.01	.03	.07	−.02	−.05	−.08	−.15
Team size	.01	.08	.09	.11	−.15	−.15	−.18	−.17
Independent variables								
Voice climate		.60^**^	.59^**^	.53^**^				
Team resilience capacity						.50^**^	.44^**^	.55**
Moderators								
Leader LGO			.10	.17				
Information elaboration							.33^*^	.32**
Interaction effects								
Voice climate × leader LGO				.31^*^				
Team resilience capacity × information elaboration								.29*
R^2^	.00	.36^**^	.37	.46^*^	.02	.27^**^	.38^*^	.44*
ΔR^2^	.00	.36^**^	.00	.09^*^	.02	.25^**^	.10^*^	.07*
F	.09	24.69^**^	25.32	32.37^*^	.56	15.73^**^	22.85^*^	27.80*
ΔF^2^	.09	24.61^**^	.62	7.06^*^	.56	15.17^**^	7.12^*^	4.96*

*Note. n* = 48. Coefficients are standardized betas. LGO = learning goal orientation. ^**^ *p* < .01, ^*^ *p* < .05.

**Figure 2.**
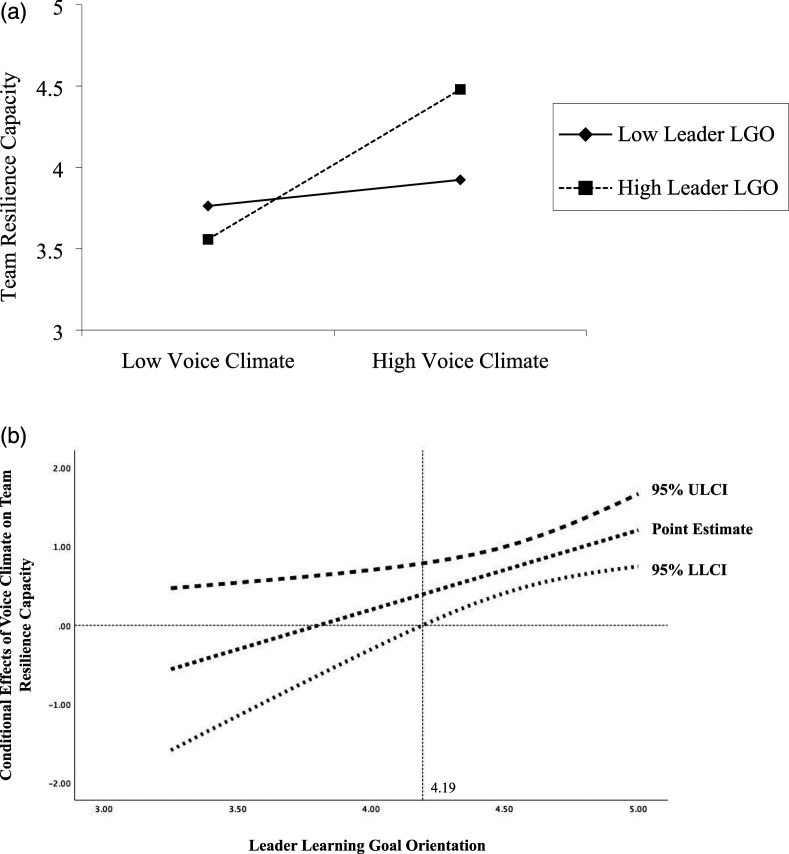
(a) Team resilience capacity as a function of voice climate and leader learning goal orientation (LGO). (b) John–Neyman regions of significance for the conditional effect of voice climate at values of leader learning goal orientation.

**Figure 3.**
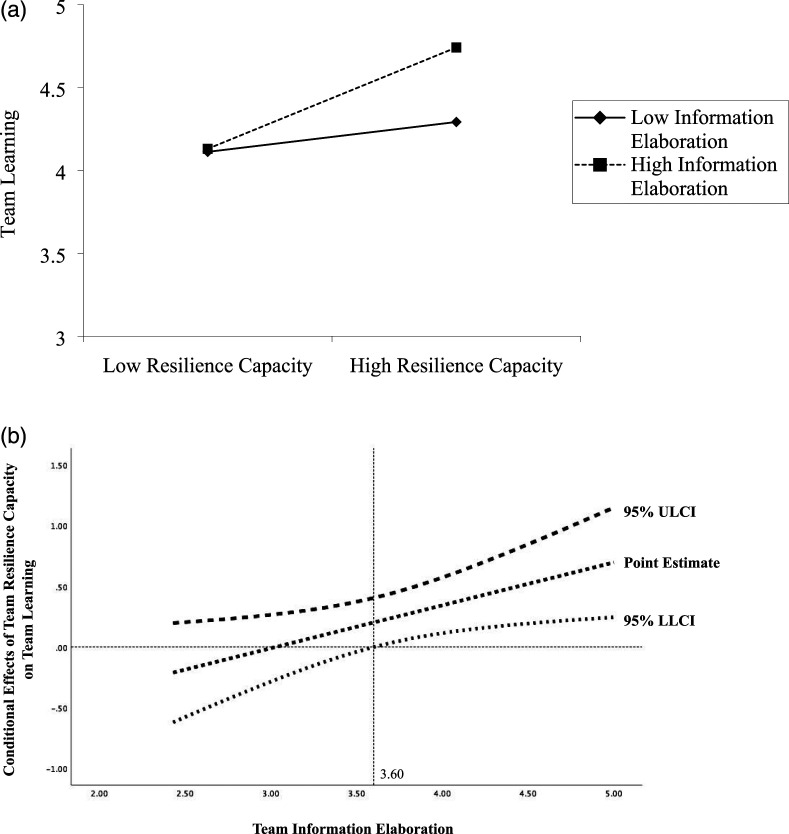
(a) Team learning as a function of team resilience capacity and information elaboration. (b) John–Neyman regions of significance for the conditional effect of team resilience capacity at values of information elaboration.

To visualize these interactions, we plotted the values of the independent variables at one standard deviation above and below the mean of the moderators, as per convention (Aiken & West, 1991), as seen in Figures 2(a) and 3(a). We also probed the significance of these conditional effects using the Johnson–Neyman technique (i.e., J–N; Hayes & Matthes, 2009; Preacher, Curran, & Bauer, 2006), as seen in Figures 2(b) and 3(b). The J–N technique has become a preferred method for assessing the significance of interactions because it identifies points along the range of the moderator where the effects of the independent variable on the dependent variable significantly differ from zero, as opposed to arbitrarily assigning a cutoff value. As illustrated in Figure 2(b), the effect of voice climate on team resilience capacity is statistically different from zero when leader LGO exceeds 4.19. As well, as illustrated in Figure 3(b), the effect of team resilience capacity on team learning is statistically different from zero when team information elaboration exceeds 3.60. That is, as we predicted, leader LGO interacted with voice climate such that teams with a high voice climate perceived themselves as even more resilient as leader LGO increased. Likewise, team information elaboration interacted with team resilience capacity such that teams with a high resilience capacity engaged in even more learning activities as information elaboration increased.

Next, we assessed Hypothesis 5 using the PROCESS macro for SPSS (Model 4; Hayes, 2017). This model estimated the indirect effects of voice climate on team learning via team resilience capacity. Results support this hypothesis, as we found that voice climate was significantly positively related to team resilience capacity (β = .60, *p* <.01) and, in turn, team resilience capacity was significantly positively related to team learning (β = .32, *p* < .05), though voice climate was not directly related to team learning (β = .30, *n.s.*). Results from the bias-corrected bootstrapping procedure for the indirect effect with 20,000 resamples at a 95% confidence interval did not include zero (β = .20; 95% CI: [.02–.46]), which suggests that voice climate is related to team learning because of its effect on team resilience capacity.

Finally, we assessed the overall model (Hypothesis 6) using PROCESS Model 21 (Hayes, 2017). In particular, we estimated the conditional indirect effect of voice climate on team learning through team resilience capacity at high and low levels of the leader LGO (stage 1) and team information elaboration (stage 2) using the bias-corrected bootstrapping procedure for the indirect effect with 20,000 resamples at a 95% confidence interval. As shown in Table 4–6, we found a significant interaction between voice climate and leader LGO on team resilience capacity (b = .70, 95% CI: [.41–1.00]), as well as between team resilience capacity and information elaboration on team learning (b = .29, 95% CI: [.08–.51]). Altogether, we found support for the full moderated-mediation model, in that both moderators amplified the effects of the independent and mediating variables along the casual chain at mean and high-levels of the moderators, consistent with Figures 2(a) and 3(a). That is, voice climate was positively related to team resilience capacity (b = .67, *p* < .01) and leader LGO moderated the effects of voice climate on team resilience capacity (b = .10, *p* = .01), while team resilience capacity was positively related to team learning (b = .28, *p* <.01) and team information elaboration moderated the effects of team resilience capacity on learning (b = .35, *p* <.05).

**Table 4.** Moderated-Mediation Results (H2: Voice Climate → Team Resilience Capacity).

Level of LGO	Level of team IE	Conditional indirect effect	SE	LLCI	ULCI
Low	—	.20	.25	−.31	.70
Med	—	.70^**^	.15	.41	1.00
High	—	1.21^**^	.23	.75	1.67

*Note. n* = 48. Coefficients are unstandardized betas. LGO = learning goal orientation; team IE = team information elaboration, LLCI = lower-level confidence interval. ULCI = upper level confidence interval; ^**^ *p* <.001.

**Table 5.** Moderated-Mediation Results (H4: Team Resilience Capacity → Team Learning).

Level of LGO	Level of team IE	Conditional indirect effect	SE	LLCI	ULCI
—	Low	.04	.12	−.21	.29
—	Med	.29^*^	.11	.08	.51
—	High	.55^*^	.17	.21	.90

*Note. n* = 48. Coefficients are unstandardized betas. LGO = learning goal orientation; team IE = team information elaboration, LLCI = lower-level confidence interval. ULCI = upper level confidence interval; ^**^ *p* <.001. ^*^ *p* <.01.

**Table 6.** Moderated-Mediation Results (H6: Voice Climate → Team Resilience Capacity → Team Learning).

Level of LGO	Level of team IE	Conditional indirect effect	SE	LLCI	ULCI
Low	Low	.01	.05	−.07	.12
Low	Med	.06	.11	−.11	.33
Low	High	.11	.20	−.19	.60
Med	Low	.03	.09	−.14	.20
Med	Med	.21	.12	.04	.49
Med	High	.39	.23	.08	.94
High	Low	.05	.15	−.24	.34
High	Med	.35	.17	.08	.75
High	High	.67	.34	.15	1.45

*Note. n* = 48. Coefficients are unstandardized betas. LGO = learning goal orientation; team IE = team information elaboration, LLCI = lower-level confidence interval. ULCI = upper level confidence interval; ^**^ *p* <.001. ^*^ *p* <.01.

## Discussion

This research responds to several recent calls for greater clarity on how resilience capacity develops in teams and what teams with a high capacity for resilience do (e.g., Duchek, 2020; Hartmann, Weiss, Newman, et al., 2020; Stoverink et al., 2020), along with the need to delineate specific boundary conditions that moderate these relationships. Results support our resource-based perspective of team resilience capacity. Specifically, we found that voice climate was positively related to team resilience capacity, and that leader LGO amplified its effect, such that teams with high voice climate, in which members felt encouraged to express voice, perceived themselves as even more capable of overcoming adversity (i.e., resilient) when they reported to a leader with a higher LGO, who pursues challenging work for personal growth. In turn, we found that team resilience capacity was positively related to team learning, and information elaboration amplified its effect, such that team with a high resilience capacity engaged in even more learning to the extent that team members shared, discussed, and integrated diverse perspectives. Altogether, reflecting back on our research questions, our results suggest that (a) voice climate builds team resilience capacity, (b) teams with higher resilience capacity engage in more learning than teams with lower resilience capacity, and (c) leader LGO amplifies the positive effects of voice climate on team resilience capacity, while team information elaboration amplifies the positive effects of team resilience capacity on team learning.

### Theoretical Implications

Our study contributes to research and theory on team resilience in several ways. First, we offer empirical support for the tenets underlying COR theory (Hobfoll, 1989) as a guiding framework to understand the emergence and function of team resilience capacity. Specifically, our results suggest that team resilience capacity develops from a caravan of critical team resources (voice climate and leader LGO) that are essential for overcoming adversity. In turn, resilient teams expend their stocks of resources to engage in learning activities, and information elaboration enhances this effect by facilitating resource exchange via “crossover.” COR theory aligns with the dominant conceptualization of team resilience as a capacity to overcome adversity, as opposed to a process or outcome of triumphing over adversity, and thus can help to unite the emerging literature on team resilience by describing how teams build a capacity for resilience through interactions that boost their reservoir of resources, which they can subsequently deploy to achieve team goals, such as by engaging in learning before, during, and after adversity strikes (Duchek, 2020; Maynard & Kennedy, 2016; Stoverink et al., 2020). As COR is grounded in stress theory (and has only recently been applied beyond that domain; see Hobfoll et al., 2018), we are excited by its potential to offer a multilevel foundation for research on team resilience in terms of how teams acquire and deploy social, cognitive, and emotional resources (Hartmann, Weiss, & Hoegl, 2020; Hobfoll et al., 2018; Stoverink et al., 2020).

We also advance research on team resilience by demonstrating its empirical links with team learning. Although learning is deeply embedded within the broader resilience literature (e.g., Sutcliffe & Vogus, 2003; Tugade & Fredrickson, 2004), it has been neglected in empirical research on team resilience, which has instead largely focused on well-being and performance outcomes (Gucciardi et al., 2018; Hartmann, Weiss, Newman, et al., 2020). Our research highlights that resilient teams engage in learning activities presumably because they are a resource-enhancing activity that helps them prepare for future challenges (e.g., minimize, Alliger et al., 2015; anticipate, Duchek, 2020). That is, our results suggest that teams with a high resilience capacity are well-positioned to engage in learning due to their abundant pool of resources. Relatedly, our finding concerning the moderating effect of team information elaboration highlights how team resilience capacity is not a panacea for all team challenges. Rather, for teams to fully capitalize on their resilience capacity, they also need social structures in place that help to mobilize team resources via efficient communication and coordination (Duchek, 2020; Hartwig et al., 2020). Coupled with the findings pertaining to leader LGO, we offer important theoretical advancements by identifying the conditions under which teams are more likely to develop resilience capacity and leverage this capacity to achieve positive outcomes.

Finally, we expand the nomological network of team resilience capacity by demonstrating its positive links with voice climate, leader LGO, information elaboration, and learning. Each of these relationships adds to our understanding of team resilience and points to intriguing future directions. First, our finding that voice climate relates to team resilience capacity supports recent evidence on how open communication and supportive environments are integral for the development of team resilience (Carmeli et al., 2013; Gomes et al., 2014; Vera et al., 2017). Furthermore, it supports the critical role of leadership in building team resilience capacity (cf. Alliger et al., 2015; Gucciardi et al., 2018) via fostering a supportive voice climate (Frazier & Bowler, 2015), which is further amplified when leaders hold a high LGO. We also advance research on voice climate by demonstrating how it facilitates important team outcomes beyond voice behavior and thus deserves greater consideration in teams research. Similarly, we advance research on goal orientation in teams, which has largely focused on team aggregate operationalizations (e.g., Chadwick & Raver, 2015), by instead illustrating how leader LGO enhances the positive relationship between voice climate and team resilience capacity.

### Practical Implications

We also offer important practical contributions by establishing which variables are essential to help teams develop the capacity needed to overcome adversity. In particular, we provide evidence suggesting *how* leaders can build their team’s resilience capacity by (a) creating a positive voice climate through active solicitation and encouragement of voice and (b) embracing an LGO focused on tackling challenging work with the goal of personal development. Overall, our results support Li and Tangirala’s (forthcoming, p. 21) contention that “voice can separate resilient teams from brittle ones,” and thus, leaders should strive to create climates that encourage voice.

Additionally, our finding regarding the moderating effect of information elaboration highlights the importance for resilient teams to leverage each member’s unique perspectives. This finding suggests that leaders would benefit from creating structures for resource mobilization and exchange to help their team fully capitalize on their resilience capacity (Chen et al., 2015; Meneghel, Martínez, & Salanova, 2016). Such structures may be especially important today, due to shifts toward distributed work triggered by COVID-19, which has created new challenges for smooth team communication and coordination (Brynjolfsson et al., 2020). In sum, we encourage organizational leaders to build team resilience capacity by emphasizing open communication and embracing an LGO through training, HRM practices, or structural changes that enable team members to freely express opinions and seamlessly integrate diverse perspectives (Bardoel et al., 2014; Bowers et al., 2017; Hobfoll et al., 2018). For example, organizations can educate leaders on the potential benefits of LGO for team resilience purposes (e.g., Vandewalle et al., 2019) or emphasize the importance of providing adequate responses when team members express voice (e.g., King, Ryan, & Van Dyne, 2019).

### Strengths, Limitations, and Future Directions

Despite these valuable contributions, our research also contains several limitations that we hope to address in future research. First, as with any model, we focused on a specific subset of antecedents, moderators, and consequences; thus, it is possible that we omitted other important variables. For example, we examined the effects of voice climate on team resilience capacity because it has been shown to facilitate information and idea sharing in teams (e.g., Frazier & Bowler, 2015) and fits our theoretical focus on team resources that enable resource acquisition and protect against resource loss (Hobfoll et al., 2015); however, it is possible that other unidentified variables, such as psychological safety, would have had a stronger influence on team resilience capacity. We encourage future research to continue exploring the nomological network of team resilience so that we can build a body of evidence concerning the relative importance of different variables. Several recent high-quality conceptual articles have identified other potential variables to explore, which we urge scholars to consider (see Alliger et al., 2015; Bowers et al., 2017; Duchek, 2020; Hartmann, Weiss, Newman, et al., 2020; Hartwig et al., 2020; Maynard & Kennedy, 2016; Stoverink et al., 2020). We also encourage researchers to explicitly define and delineate their conceptualization of team resilience to offer greater precision as to whether they are examining team resilience as a capacity, process, or outcome.

Second, we measured variables across time and with different respondents to proactively address concerns of common-method bias by introducing temporal precedence. One exception, however, is that we assessed team members’ perceptions of voice climate and team resilience capacity at the same timepoint, which introduces two potential issues. First, this relationship may be inflated by a common method. Results of a supplemental confirmatory factor analysis support the discrimination of these constructs, as a model with both factors separated (*X*^2^[26] = 77.21, CFI = .95, RMSEA = .10, SRMR = .04) fits significantly better than a model with factors combined (*X*^2^[27] = 351.39, CFI = .71, RMSEA = .24, SRMR = .10). Nevertheless, we cannot rule out the possibility that common-method bias affected their relationship. At the same time, it is important to clarify that common-method bias is a linear phenomenon, and thus, it does not affect moderation results (Siemsen, Roth, & Oliveira, 2010). The second issue is that we cannot establish that voice climate *causes* team resilience capacity because temporal precedence is a necessary precondition for causal conclusions (Mathieu et al., 2008; Spector, 2019). However, as described below, this issue is endemic to all survey methods examining conditions or experiences with teams that existed prior to data collection, in which case temporally separating the measurement of voice climate and team resilience capacity would still not enable causal interpretations, even if it offers “face validity.” Finally, it is important to clarify that this cross-sectional approach only affects part of our model and is regarded as appropriate when theory supports the predicted relationship (Mathieu et al., 2008), particularly for relationships that have not been identified in prior research (Spector, 2019). Nevertheless, we encourage future research to continue to explore, expand, and refine our model to offer greater evidence of causality, such as by measuring the variables at multiple timepoints and probing for potential alternative explanations.

Relatedly, we positioned team resilience capacity as antecedent to learning based on the notion that team states precede behaviors (Mathieu et al., 2008). Although we measured team resilience capacity prior to team learning, our results do not infer causality because we cannot speak to the team conditions that existed prior to measurement, as discussed above (Spector, 2019). We also noted that resilience capacity is a dynamic team property, but we measured it at one timepoint, and thus cannot assess changes in team resilience capacity over time. Therefore, it is possible that some teams felt more resilient because they previously engaged in learning activities, which our methods could not assess. Future research would benefit from measuring these constructs longitudinally, with new teams, or with experimental methods to tease apart causal effects. For example, given the likely reciprocal links between team resilience and learning, it would be interesting for scholars to experimentally manipulate team resilience capacity, such as by providing teams with negative feedback such that it diminishes their collective perceptions of resilience, and subsequently chart their capacity to engage in learning. Overall, we suspect that team resilience exhibits a recursive relationship with learning, such that resilient teams are well-suited to engage in learning to prepare for challenges, just as learning provides resilient teams with resources to overcome future adversities. This perspective aligns to recent considerations in the literature regarding how resilient teams respond before, during, and after adversity strikes, such as by monitoring and exchanging information about potential challenges beforehand (i.e., minimize and anticipate) and evaluating challenges afterward via debriefs (i.e., mend and adapt; Alliger et al., 2015; Duchek, 2020); however, we focus on the direction from resilience to learning in this article to provide an initial empirical perspective on their relationship.

Our results may also have been influenced by the unique sample of knowledge-intensive and task-interdependent teams operating in emerging start-ups. For example, Sanner and Bunderson (2015) provide meta-analytic evidence that knowledge-intensity moderates the effects of psychological safety on team learning. Thus, it is possible that voice climate may be especially relevant for building team resilience capacity, and team resilience capacity for mediating its effects on team learning, for teams working on complex and creative tasks that require knowledge exchanges, as was typical of our sample. At the same time, it is important to note that resilience is relevant and useful to any occupation (Kossek & Perrigino, 2016); thus, these mechanisms seem relevant to a wide range of teams. Finally, our model details a primarily cognitive process of resilience and thus overlooks how emotions spread within teams to influence the development and consequences of resilience capacity. For example, voice climate may also affect team resilience capacity by reducing fears of punishment (e.g., Kish-Gephart, Detert, Treviño, & Edmondson, 2009). Indeed, several scholars have described the links between team affect and resilience (e.g., Meneghel, Salanova, & Martínez, 2016; Stephens et al., 2013). Thus, we encourage future research to consider cognitive and affective mechanisms in tandem to fully understand how team resilience capacity develops and subsequently affects team functioning. Overall, we view our study as a launching point for research and theory on team resilience as we clarify some of the foundations and consequences of team resilience capacity, along with the boundary conditions under which we are more likely to observe these effects.

## Conclusion

Grounded in COR theory, we present and demonstrate support for a model that links a specific team resource, voice climate, to a critical team output, learning behaviors, via team resilience capacity. In addition, we identify leader LGO as an important mechanism that activates and amplifies the role of voice climate in facilitating team resilience capacity and team information elaboration as a critical mechanism that enhances the positive effect of team resilience capacity on team learning. This work answers the calls of scholars to empirically uncover states and resources that facilitate team resilience, demonstrate key outcomes of team resilience, and detail boundary conditions of resilience effects. It is our hope that the precision employed in the conceptualization and operationalization of team resilience capacity in our work contributes to clarity within this domain and that future work will continue to build upon the team resilience nomological network extensions offered here.

## Notes

1 While similar to the Input-Mediator-Output-Input model of teamwork (IMOI; Ilgen, Hollenbeck, Johnson, & Jundt, 2005), COR is an explanatory theory that describes *how* and *why* teams acquire and subsequently expend resources to achieve goals. By contrast, IMOI is a general organizing framework intended to guide research on the mechanisms linking team inputs to outputs and thus does not offer the same theoretical precision.

2 Morrison et al. (2011) also conducted factor analysis to demonstrate that voice climate is empirically distinct from psychological safety.

3 We determined that a 2-week time lag was appropriate to create a temporal separation between the constructs, but not too long that it masks true relationships (Dormann & Griffin, 2015; Podsakoff et al., 2003, 2012). We were also worried that a longer lag would introduce irreparable logistical issues (e.g., changes to team membership).

4 Team leaders were not included as team members. None of the teams in our study reported to the same leader, though two teams had two leaders, in which case we computed leader ratings as a mean between both leaders.

5 We determined that leaders could accurately evaluate their team’s learning and information elaboration behaviors because they have unique knowledge regarding these behaviors (Kozlowski & Klein, 2000). Moreover, our measure of team learning (Edmondson, 1999) was developed specifically for observer ratings, while research on team information elaboration generally relies on observer ratings (e.g., Homan et al., 2007; Resick et al., 2014).
